# Mycolactone-Dependent Depletion of Endothelial Cell Thrombomodulin Is Strongly Associated with Fibrin Deposition in Buruli Ulcer Lesions

**DOI:** 10.1371/journal.ppat.1005011

**Published:** 2015-07-16

**Authors:** Joy Ogbechi, Marie-Thérèse Ruf, Belinda S. Hall, Katherine Bodman-Smith, Moritz Vogel, Hua-Lin Wu, Alexander Stainer, Charles T. Esmon, Josefin Ahnström, Gerd Pluschke, Rachel E. Simmonds

**Affiliations:** 1 Department of Microbial and Cellular Sciences, School of Biosciences and Medicine, Faculty of Health and Medical Sciences, University of Surrey, Guildford, United Kingdom; 2 Department of Medical Parasitology and Infection Biology, Swiss Tropical and Public Health Institute, Basel, Switzerland; 3 University of Basel, Basel, Switzerland; 4 Section Clinical Tropical Medicine, Heidelberg University Hospital, Heidelberg, Germany; 5 Department of Biochemistry and Molecular Biology, National Cheng Kung University, Tainan, Taiwan; 6 Institute for Cardiovascular and Metabolic Research, School of Biological Sciences, University of Reading, Reading, United Kingdom; 7 Coagulation Biology Laboratory, Oklahoma Medical Research Foundation, Oklahoma City, Oklahoma, United States of America; 8 Centre for Haematology, Faculty of Medicine, Imperial College London, London, United Kingdom; McGill University, CANADA

## Abstract

A well-known histopathological feature of diseased skin in Buruli ulcer (BU) is coagulative necrosis caused by the *Mycobacterium ulcerans* macrolide exotoxin mycolactone. Since the underlying mechanism is not known, we have investigated the effect of mycolactone on endothelial cells, focussing on the expression of surface anticoagulant molecules involved in the protein C anticoagulant pathway. Congenital deficiencies in this natural anticoagulant pathway are known to induce thrombotic complications such as *purpura fulimans* and spontaneous necrosis. Mycolactone profoundly decreased thrombomodulin (TM) expression on the surface of human dermal microvascular endothelial cells (HDMVEC) at doses as low as 2ng/ml and as early as 8hrs after exposure. TM activates protein C by altering thrombin’s substrate specificity, and exposure of HDMVEC to mycolactone for 24 hours resulted in an almost complete loss of the cells’ ability to produce activated protein C. Loss of TM was shown to be due to a previously described mechanism involving mycolactone-dependent blockade of Sec61 translocation that results in proteasome-dependent degradation of newly synthesised ER-transiting proteins. Indeed, depletion from cells determined by live-cell imaging of cells stably expressing a recombinant TM-GFP fusion protein occurred at the known turnover rate. In order to determine the relevance of these findings to BU disease, immunohistochemistry of punch biopsies from 40 BU lesions (31 ulcers, nine plaques) was performed. TM abundance was profoundly reduced in the subcutis of 78% of biopsies. Furthermore, it was confirmed that fibrin deposition is a common feature of BU lesions, particularly in the necrotic areas. These findings indicate that there is decreased ability to control thrombin generation in BU skin. Mycolactone’s effects on normal endothelial cell function, including its ability to activate the protein C anticoagulant pathway are strongly associated with this. Fibrin-driven tissue ischemia could contribute to the development of the tissue necrosis seen in BU lesions.

## Author Summary

Buruli ulcer (BU) is a neglected tropical disease that is most common in West Africa and parts of Australia, but has been reported from over 30 countries worldwide. The symptoms are painless ulcers of the skin caused by a bacterial infection. The bacteria, *Mycobacterium ulcerans*, produce a macrolide toxin called mycolactone. In this manuscript, we have studied the effects of mycolactone on endothelial cells, specialised cells that line blood vessels and form capillaries. One of the most important functions of these cells is to prevent blood from clotting inside the vessels. We show that mycolactone reduces the ability of cultured endothelial cells to anticoagulate blood, by blocking the expression of a protein called thrombomodulin. We went on to examine samples of BU patient skin and found that thrombomodulin is also reduced here, and that in contrast to normal skin large amounts of fibrin (one of the main constituents of blood clots) were present. This means that it may be useful to consider whether anticoagulants might improve the response to antibiotics and thereby improve treatment outcomes for BU patients.

## Introduction

The neglected tropical disease Buruli ulcer (BU), caused by subcutaneous infection with *Mycobacterium ulcerans*, is most common in West Africa, but has also reported from Australia and altogether more than 30 countries worldwide [1]. These indolent, painless, ulcers affect at least 5,000 patients/year and are thought to be heavily under-reported. Without treatment, ulcers can extend to 15% of the skin surface area and cause significant morbidity [2]. The route of transmission of the infection is still controversial with proposed routes spanning from biting water insects to passive entry from an environmental reservoir via physical damage to skin [3,4]. The disease may present in different forms including indurated subcutaneous nodules, plaques, ulcers and oedema. The WHO recommended standard treatment consists of 8 weeks combination antibiotic therapy with streptomycin and rifampicin [5,6]. Adjunct surgical debridement followed by skin grafting is often required for larger lesions [7–9]. The specific treatment of the *M*. *ulcerans* infection remains burdened by the long duration, route of application and potential toxicity of the current drug regimen. The adjunct surgery suffers from a lack of skilled personnel, material supplies and inadequate wound care. As such, a therapeutic approach reducing the lesion size could profoundly improve the status quo in BU care.

Much insight into the pathogenesis and treatment of BU has been gained by studying the histopathology of infected tissues [9–11]. Strikingly, lesions show large clusters of extracellular acid-fast bacilli, extensive necrosis, and a relative lack of infiltrating immune cells [12]. All of these features are attributed to a lipid-like virulence factor produced by *M*. *ulcerans* called mycolactone [13]. Mycolactone is detected at high levels within ulcers [14,15], but pathology associated with its production is also evident some distance away from the microbes. The toxin may even cause some systemic immunosuppression [14]. Mycolactone purified from the acetone-soluble lipids of *M*. *ulcerans* was first described in 1999 [13]. It was subsequently shown that it can recapitulate the effects of the microbe, since injection of 100μg mycolactone in guinea pig skin caused ulceration within 5 days [13] with pathological features similar to those caused by *M*. *ulcerans* infection. Mycolactone causes apoptosis within the skin of infected guinea pigs, as revealed by TUNEL staining, in primary human keratinocytes [16] and also in cultured L929 and J774 cells which undergo cytoskeletal rearrangement and round up [17]. By 48 hours most of the cells have completely detached from the tissue culture plate, although these cells are still viable up to several days after they detach [17]. Nevertheless, mycolactone causes cell cycle arrest in G_0_/G_1_ stage of the cell cycle [13], and mycolactone treated cells are known to display growth inhibition [13,18].

The extensive tissue necrosis and cell death seen in BU would normally be expected to serve as a trigger for an inflammatory response in addition to that induced by the invading bacteria itself. However, the reverse is the case as there is no pain and little inflammation even in the presence of high bacterial loads in the centre of the lesions [1]. Mycolactone has been demonstrated to have a suppressive effect on the cells of both the innate and adaptive immune system, including dendritic cells, monocytes and T lymphocytes [19–23], preventing an effective immune response against the infection. Successful antibiotic treatment has been shown to be associated with a strong local immune response, with large numbers of infiltrating mononuclear cells and granuloma formation at the site of infection [10]. A possible mechanism may be cessation of mycolactone production prior to sterilisation, allowing the body’s own defences to play a role in healing [15].

Despite this wealth of research described above, to date there has been no molecular explanation of the coagulative necrosis seen in the ulcers beyond the cytopathic activity of mycolactone or its immunosuppressive effects, nor any description of the effects of mycolactone on endothelial cells. We recently showed that mycolactone is an inhibitor of Sec61-dependent translocation, thereby preventing the production of new secreted and membrane proteins that transit through the ER [18], including in these cells. We were intrigued as to how coagulative necrosis, and reports of clots and fibrin deposition evident within BU-associated vessels [24,25] could be linked to this activity and postulated that mycolactone may reduce the ability of endothelial cells to inhibit clot formation.

Endothelial cells form a highly dynamic organ that, together, regulate many physiological functions, and crucial to these is the regulation of coagulation [26]. One of the ways this is achieved is by the expression of specific receptors that together activate the protein C anticoagulant pathway [27]. When thrombin binds to thrombomodulin (TM), it ceases to be a procoagulant and instead becomes a potent anticoagulant, activating protein C which can then go on to inactivate factors Va and VIIIa together with its cofactor protein S, thereby down-regulating thrombin production over the endothelial cell surface. A second receptor, the endothelial cell protein C receptor (EPCR), enhances this and mediates the cytoprotective effects of activated protein C (APC) [28]. The microvasculature of the skin expresses both TM and the EPCR [29].

In this manuscript we have investigated the effect of mycolactone on these anticoagulant endothelial receptors, the molecular basis of depletion and the ability of mycolactone-exposed endothelial cells to activate protein C. Since our findings show that TM expression is highly sensitive to mycolactone, we determined its abundance in punch biopsies from BU lesions, proving that our findings are relevant *in vivo*. Indeed, we also found that fibrin deposition is a common feature of untreated BU lesions.

## Results

### Mycolactone causes depletion of protein C receptors from the surface of primary human dermal microvascular endothelial cells

**Fig 1:**
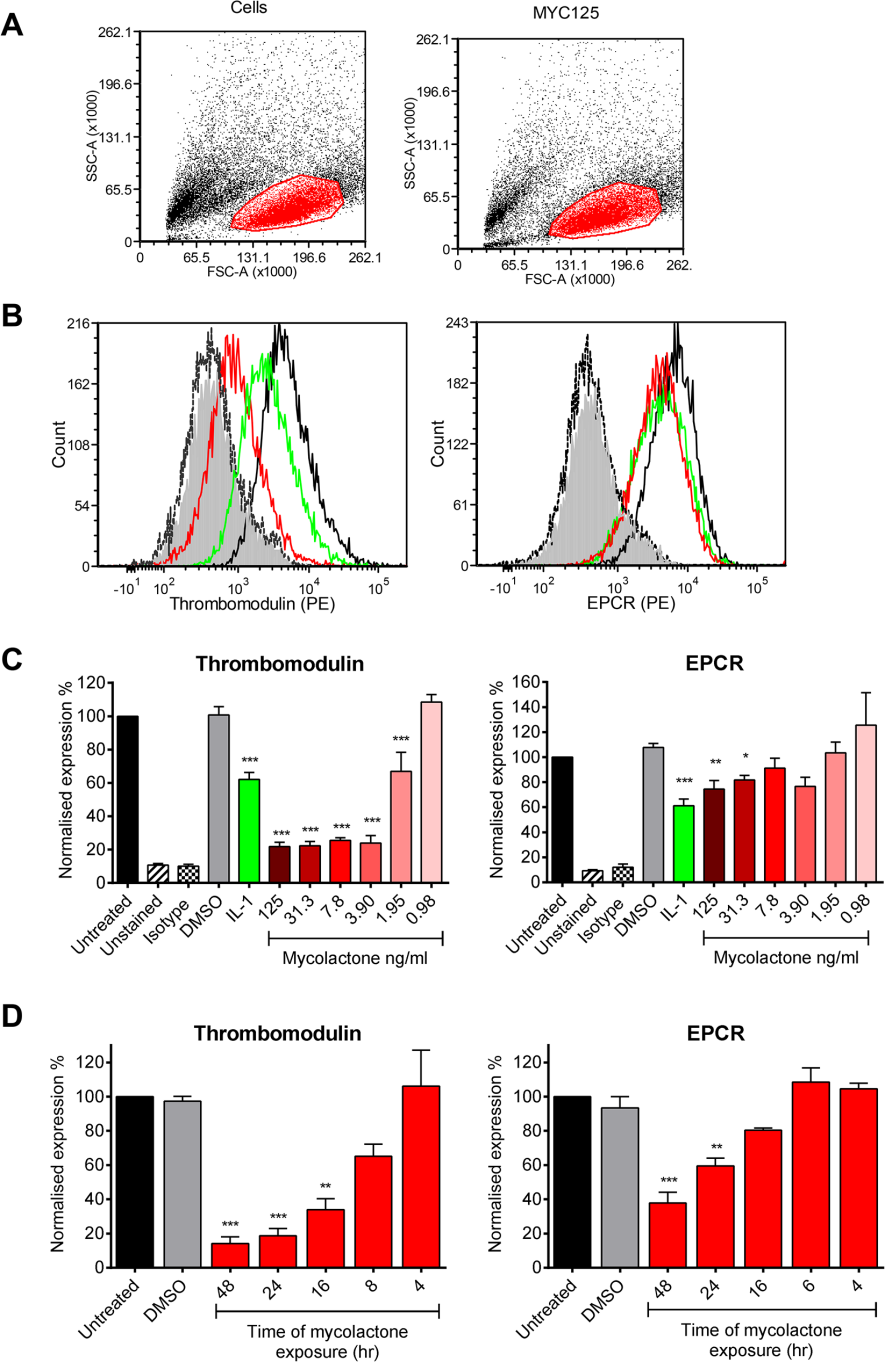
Mycolactone causes a profound depletion of thrombomodulin (TM) endothelial cell surfaces. Human dermal microvascular endothelial cells were exposed to various concentrations of mycolactone (MYC), 10ng/ml IL-1β or 0.025% DMSO (solvent control, equivalent to that in 125ng/ml MYC) for 24 hours (or other times as indicated). Cells were harvested and subjected to flow cytometry for TM and EPCR. A. Forward and side scatter plots. B. Histogram plots for TM and EPCR. Untreated unstained cells; filled grey, untreated cells with isotype control; dotted black line, untreated cells with TM or EPCR-PE; black line; MYC treated cells (7.8ng/ml) with TM or EPCR-PE, red line; IL-1β treated cells with TM or EPCR-PE, green line. C. Quantitation of TM and EPCR surface expression. MFIs are expressed as % of untreated cells with TM or EPCR-PE (mean±SEM, n = 3). Unstained and isotype bars are for untreated cells. D Quantitation of TM and EPCR surface expression over time in the presence of 7.8ng/ml mycolactone. MFIs are expressed as % of untreated cells with TM or EPCR-PE. DMSO control (0.08%) is after 48hrs exposure (mean±SEM, n = 3). *, P<0.05; **, P<0.01; ***, P<0.001.

Since the coagulative necrosis is primarily seen in the subcutaneous layer of the skin that contains abundant pervasive microvasculature, we have used primary human dermal microvascular endothelial cells to investigate the effect of mycolactone on the anticoagulant functions of the capillaries. Using flow cytometry we quantified expression of TM and the EPCR on the surface of the cells after 24hrs of exposure to 0.98–125ng/ml synthetic mycolactone A/B (Fig 1), which did not alter the physical characteristics of the exposed cells (Fig 1A). In line with previous observations [30,31], the proinflammatory cytokine IL-1β reduced the expression of TM by ~40% whereas DMSO (the mycolactone solvent [18]) had no effect (Fig 1B and 1C). Mycolactone dose-dependently and profoundly depleted the expression of TM, resulting in fluorescence levels 80% lower than solvent exposed cells at doses as low as 3.9ng/ml (Fig 1B and 1C). This same dose of mycolactone A/B could not prevent cytokine production by RAW264.7 cells [18], reinforcing the view that different types of cells display profoundly different sensitivities to mycolactone. Expression of the EPCR was also inhibited by mycolactone exposure, but a higher dose (31.3ng/ml; Fig 1C) was required for a statistically significant effect and the maximum inhibition was ~30%.

Based on this titration of mycolactone, a dose of 7.8ng/ml mycolactone was chosen to investigate the time dependence of protein depletion. Loss of TM increased over time (T_½_ 11.7±1.4hrs) and reached statistical significance at 16hrs of mycolactone exposure (Fig 1D). We also observed a time-dependent decrease in EPCR abundance, and although the rate and extent of depletion was slower and smaller than TM (Fig 1D), the cells had still lost ~60% of their EPCR by 48hrs.

### The mechanism of thrombomodulin depletion from endothelial cells is driven by the translocation-blocking activity of mycolactone

**Fig 2:**
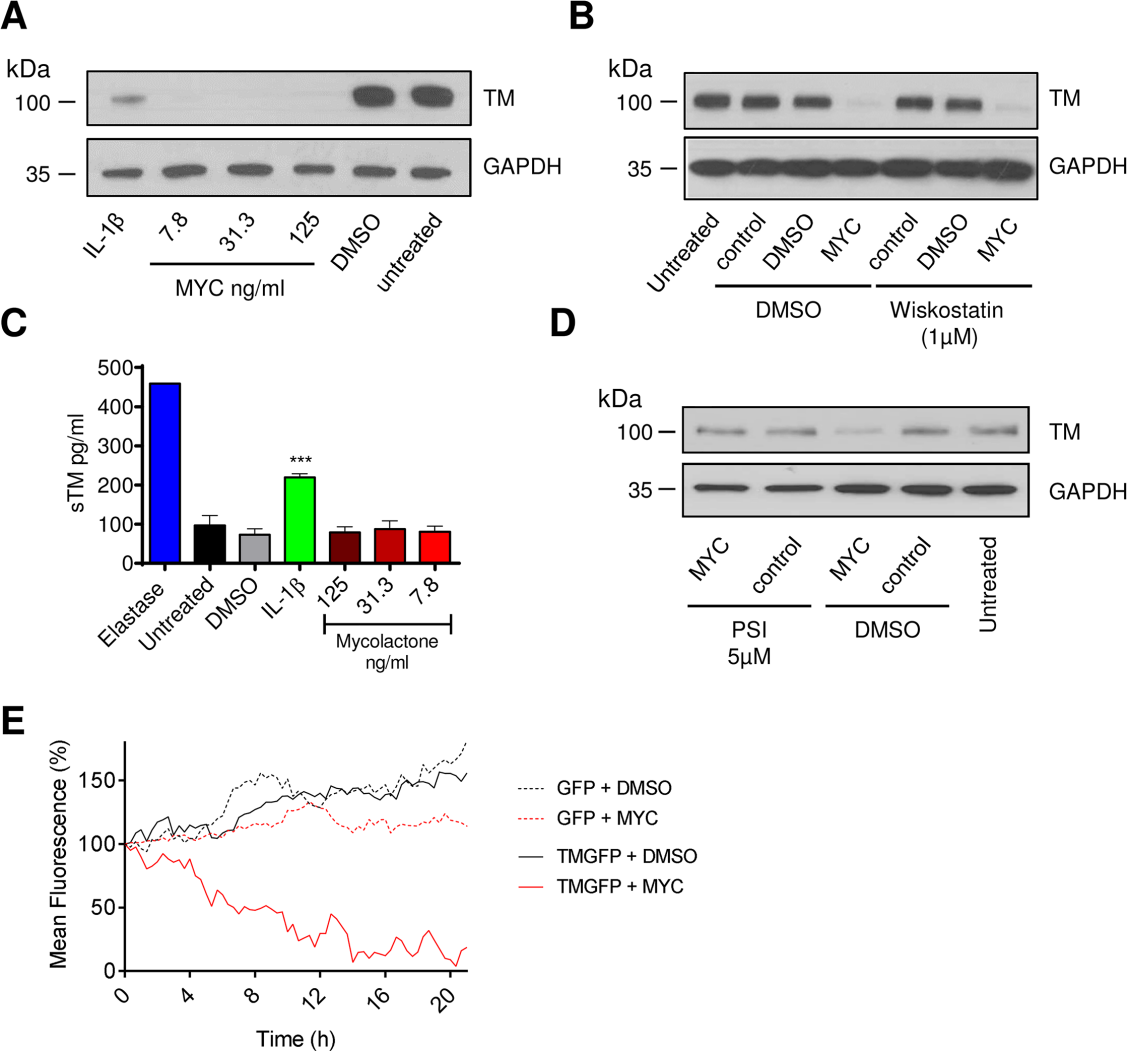
The mechanism of thrombomodulin (TM) loss on endothelial cell surfaces. Human dermal microvascular endothelial cells were exposed to various concentrations of mycolactone (MYC), 10ng/ml IL-1β or 0.08% DMSO (solvent control) for 24 hours. A. Cells were lysed and subject to Western blot analysis. B. Cells were treated as above in the absence or presence of wiskostatin (1μM), harvested and subject to Western blot analysis. C. Cells were treated as above or with 5μg/ml N-elastase. Supernatants of cells were collected, clarified by centrifugation and sTM was quantified by ELISA (mean±SEM, n = 3, except for elastase where n = 1). D. Cells were treated as above in the absence or presence of protease inhibitor I (PSI, 5μM), harvested and subject to Western blot analysis. E. HeLa cells were stably transfected with a plasmid encoding either GFP alone (expressed in the cytosol) or TM-GFP (a C-terminal fusion of human TM and GFP, therefore expressed on the membrane in an ER-dependent manner). These cell lines were subsequently exposed to 125ng/ml mycolactone or 0.025% DMSO over 21 hours and fluorescence was captured by time-lapse microscopy using a Nikon A1 confocal laser scanning unit attached to an Eclipse Ti microscope. Whole-field fluorescence is expressed as a percentage of starting fluorescence for each of triplicate fields and is expressed as the mean (corrected for background fluorescence).

Loss of TM was not restricted to surface protein, rather total cellular abundance was reduced (Fig 2A). We also examined whether the Wiskott–Aldrich syndrome protein (WASP) inhibitor wiskostatin could restore TM expression [32], but could find no effect, suggesting that a change in actin dynamics does not contribute to the decreased abundance (Fig 2B). Furthermore, while elastase caused soluble TM (sTM) to be released from the cells as expected [33], no such increase could be observed following mycolactone treatment (Fig 2C). This was in contrast to IL-1β treatment, where at least part of the loss of cell surface TM could be attributed to shedding (Fig 2C). However, similarly to Cox-2 and TNF in macrophages [18], TM depletion could be reversed by inhibiting the proteasome (Fig 2D).

We also carried out time-lapse live fluorescence microscopy of HeLa cells (Fig 2E and S1–S4 Videos). The cells stably expressed recombinant TM as an in-frame fusion protein with carboxy-terminal GFP ("TM-GFP"). Production and membrane expression of this protein, like endogenous TM, was dependent on its co-translational translocation into the ER. As a control we used cells stably expressing recombinant GFP alone ("GFP") produced in the cytosol and thus not dependent on translocation. Exposure of cells to 125ng/ml mycolactone caused ER-transiting membrane TM-GFP (S2 Video and Fig 2E solid red line), but not cytosolic GFP (S4 Video and Fig 2E dotted red line), to undergo a gradual reduction in the cell-surface fluorescence of each cell (T_½_ 8.5±0.6hrs, Fig 2E). Exposure of cells expressing TM-GFP to DMSO did not cause the same effect (S1 Video and Fig 2E solid black line). Taken together our data strongly support a mechanism of TM loss involving Sec61-dependent translocation blockade and consequent degradation of mislocated TM in the cytosol [18].

It is also interesting to note that the mean whole-field fluorescence of cytosolic GFP-expressing cells exposed to mycolactone did not increase similarly to DMSO exposed cells (Fig 2E, compare GFP + DMSO [dotted black line] with GFP + MYC [dotted red line]). This is a clear demonstration of the growth inhibition caused by mycolactone [13,18] that prevents normal cell division and associated increase in whole-field fluorescence (S3 Video). The effect of mycolactone on the adhesion of these cells can also be clearly seen; over the 20hr experiment, the cells became increasingly rounded. Notably, mycolactone-exposed cells were apparently still able to form lamellipodia, but these structures were unable to result in functional attachment to the tissue culture well surface (see S4 Video).

### Mycolactone has no direct effect on thrombin generation or platelet function, but causes a profound inhibition of endothelial cells’ ability to activate protein C

**Fig 3:**
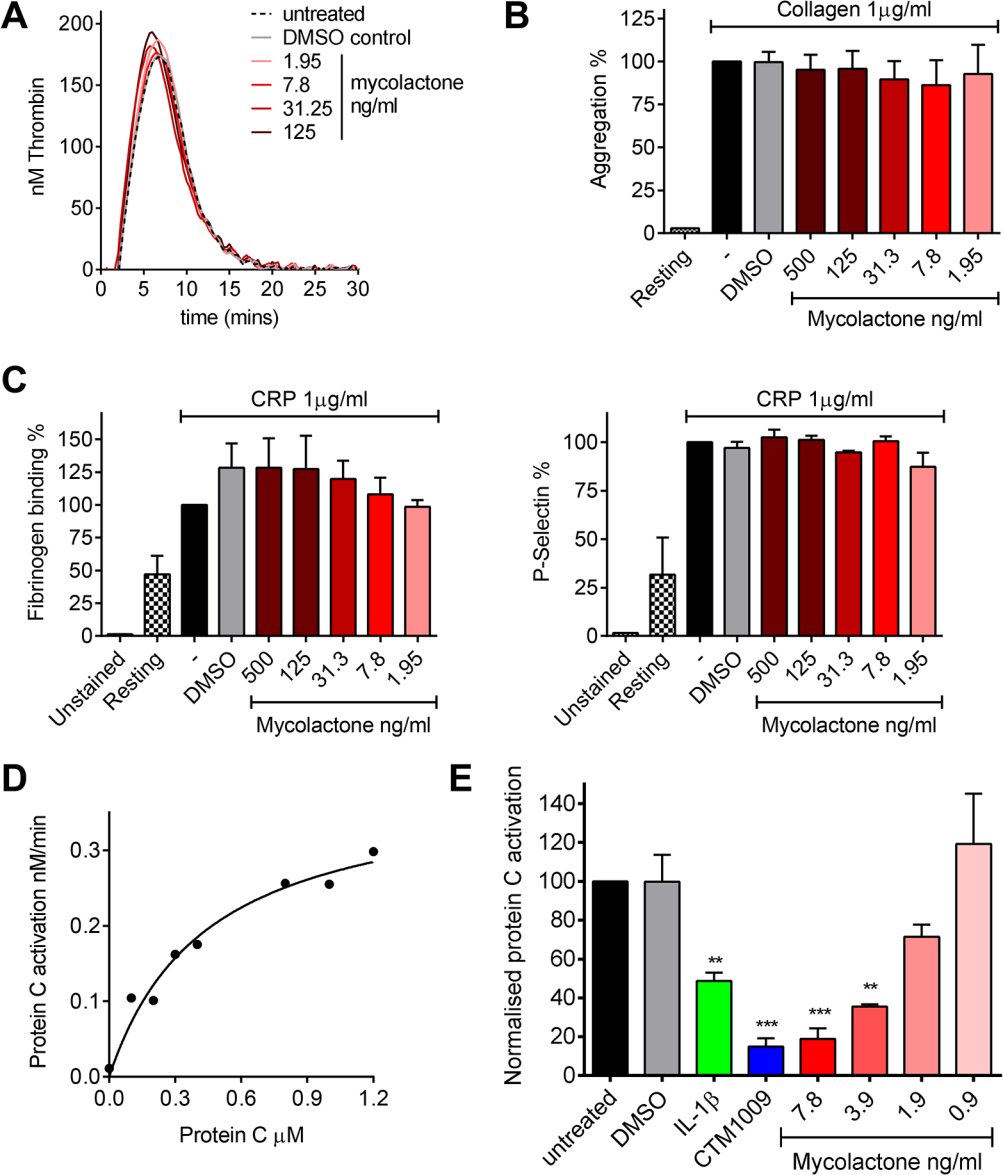
Mycolactone does not affect thrombin generation *per se* but profoundly inhibits the ability of endothelial cells to activate protein C. A. Thrombin generation was measured by calibrated automated thrombography. Thrombin generation was quantified in human pooled plasma containing various concentrations of mycolactone or 0.013% DMSO as a control. The experiment was initiated with 4pM tissue factor, 4μM phospholipid vesicles, and 16.6mM CaCl_2_. Thrombin generation was monitored using 0.42mM of the fluorogenic substrate Z-GlyArg-AMC-HCl as described in the text. B. Platelet aggregation was assessed using an optical platelet aggregometer by the addition of 1μg/ml collagen to washed human platelets that had been treated with various concentrations of mycolactone or 0.1% DMSO as a control, and are expressed relative to an untreated control. Mean±SEM n = 3 (except for resting platelets where n = 1). C. Platelet activation was determined by quantifying fibrinogen binding to, and P-selectin exposure on, human platelets by flow cytometry. Washed human platelets were treated with various concentrations of mycolactone or 0.1% DMSO as a control then stimulation with 1μg/ml CRP-XL, and are expressed relative to an untreated control. Mean±SEM n = 3. D and E. Protein C activation over human dermal microvascular endothelial cells. Protein C was added to cells in the presence of Ca^2+^ and Mg^2+^. Activation was initiated by the addition of 13.5nM thrombin and proceeded for 30 mins at which point the reaction was stopped 5μg antithrombin and 3U heparin. Activated protein C was quantified by assessing the rate of chromogenic substrate S2366 cleavage compared to an APC standard curve. D. Michaelis-Menton curve of protein C activation. E. Cells were exposed to various concentrations of mycolactone (MYC), 10ng/ml IL-1β or 0.025% DMSO (solvent control) for 24 hours. In one case, untreated cells were exposed to a function-blocking antibody (CTM) for 1hr prior to the assay. Protein C (5nM) activation was assessed as before and is expressed as % of untreated cells. Mean±SEM n = 3.

TM is an essential protein that converts thrombin from a pro- to an anti-coagulant role in the protein C anticoagulant pathway, so we went on to investigate any direct or indirect effects of mycolactone on coagulation. The coagulation cascade is dependent on Ca^2+^-dependent interactions of the Gla domains of γ-carboxylated proteins with negatively charged phospholipids and activated platelets, and given mycolactone’s hydrophobic lipid-like nature [13] it seemed possible that it might directly alter this process. We used calibrated automated thrombography (CAT) to investigate this but could find no changes in the rate of thrombin generation or lag phase at any dose of mycolactone tested (Fig 3A), or even at doses as high as 0.67μg/ml (S1A Fig). We also investigated whether mycolactone might alter the function of platelets. Mycolactone did not alter the aggregation of platelets in response to collagen (Fig 3B), or alone (S1B Fig). Similarly it did not alter fibrinogen binding or P-selectin exposure after activation by collagen-related peptide (CRP, Fig 3C), or alone (S1B Fig).

On the other hand, mycolactone did profoundly block the ability of HDMVEC to activate protein C. First we estimated the apparent K_m_ for protein C activation on these cells according to a previously optimised method [34,35] (Fig 3D), which was 444nM, compatible with the literature. We then went on to compare the rate at which 0.5μM protein C was activated over cells treated with mycolactone. The specificity of this assay for TM-dependent activation was determined using the function-blocking antibody CTM1009, which prevents thrombin binding to TM [36]. This resulted in an ~80% reduction in the amount of activated protein C (APC) formed (Fig 3E). IL-1β treatment reduced APC generation, as expected and, strikingly, 7.8ng/ml mycolactone reduced APC generation almost to the same extent as CTM1009 (~75%). Even 1.9ng/ml mycolactone reduced APC generation ~20% in the 30 minute activation assay. If a similar depletion of TM could be demonstrated in endothelial cells of BU lesions, then the vessels they line would be severely impeded in their ability to anticoagulate the circulating blood.

### Thrombomodulin is greatly reduced in the infected skin of patients with Buruli ulcer

**Fig 4:**
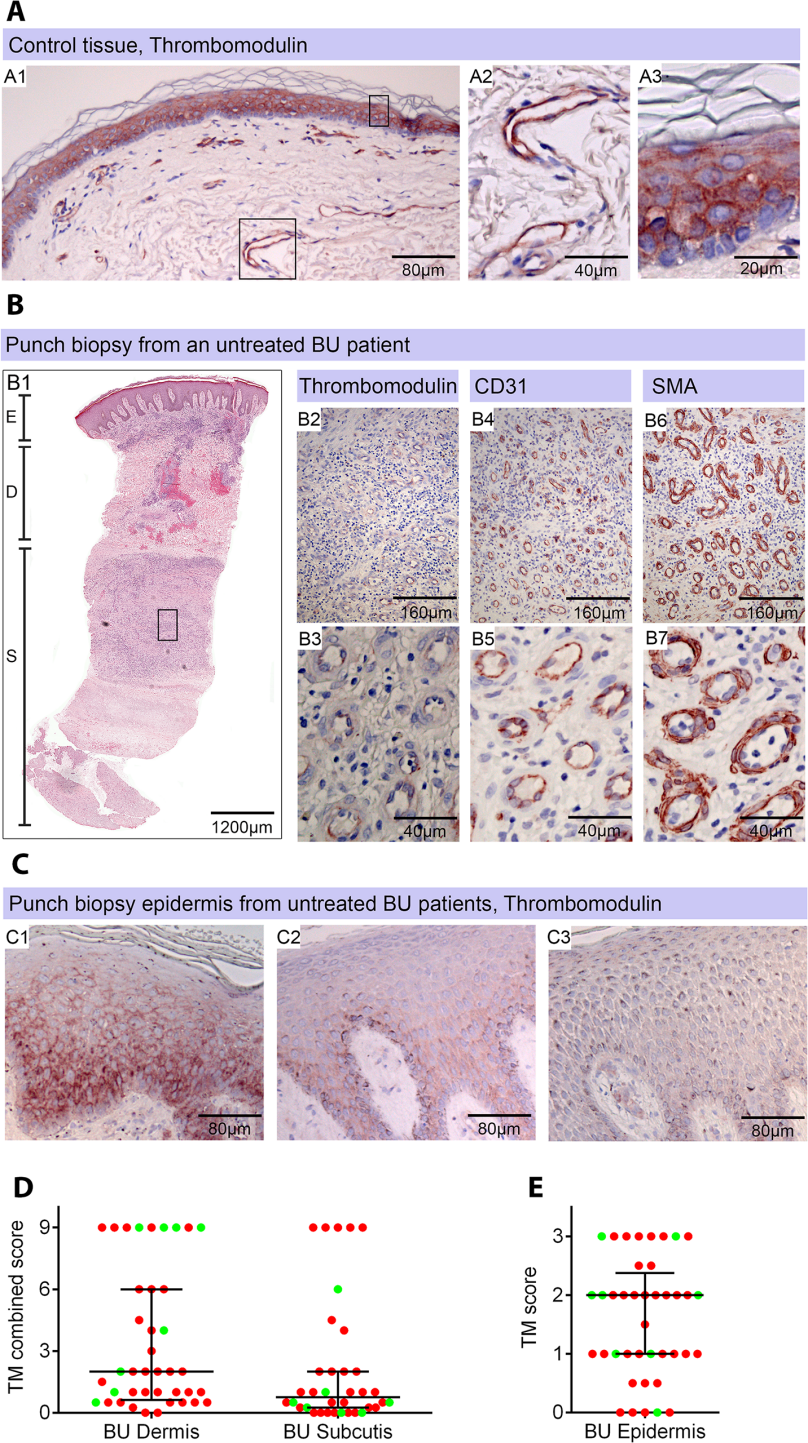
Thrombomodulin (TM) expression is dysregulated in Buruli ulcer patient skin and tissue. Histological sections were stained with α-TM, α-CD31 (PECAM-1) or α-SMA (smooth muscle actin) antibodies and counterstained with Haematoxylin or with Haematoxylin-Eosin (B1). Slides were analyzed with a DM2500 Microscope (Leica). Pictures were taken either with an Aperio scanner (B1) or a Leica DFC 420 camera and the Leica application Suite V4 software. Comparative staining on healthy skin sample from an unaffected donor (A) or 4mm punch biopsies from laboratory confirmed BU patients (B and C). Typical results are shown. A. TM staining of healthy skin. A1, Low magnification image; A2 and A3, higher magnification showing strong TM staining of endothelial cells and keratinocytes, respectively. B1. Scan of a HE stained BU punch biopsy (E; Epidermis, D; Dermis, S; Subcutis). B2 –B7, higher magnification of subcutaneous tissue showing reduced TM staining in the endothelium (B2 and B3). This patient had a combined score of 2 in the dermis (coverage 2, intensity 1) and 2 in the subcutis (coverage 2, intensity 1). Endothelial cells in the region still showed reasonable staining for CD31 (B4 and B5) and strong staining for αSMA (B6 and B7), scoring 2.5 in the dermis and 3 in the subcutis. C. Higher magnification of the epidermis showing variable reduction in TM staining in the keratinocytes of BU patient skin, ranging from reduced (C1) to no staining (C3). Hyperplasia of the epidermis, as seen in these three patients, is typical of BU. D and E. TM staining in the dermis, subcutis and epidermis was scored as described in Materials and Methods. The score for each individual biopsy analysed is shown, consisting of 31 patients with ulcers (red circles) and 9 patients with plaque lesions (green circles). In all cases, error bars show the median and 25–75% percentile scores for the all BU patients. D. Scores of dermis and subcutis are for endothelial TM and were obtained by multiplying two scores (each 0–3) of intensity of staining and the coverage, (healthy tissue scored the maximum 9). Numbers for subcutical staining are slightly lower since those patients that had no intact endothelium in the subcutis according to SMA staining were excluded. E. Score for epidermal TM (keratinocytes) are relative to a maximum of 3 (intensity only).

Healthy skin is a highly heterogeneous tissue composed of diverse tissue types and, in BU, a wide variation in histopathological changes is seen in lesions of the same category. Therefore, in order to establish the *in vivo* relevance of these findings, the abundance of TM in 4mm punch biopsy samples taken from BU lesions was assessed, using the delberately cautious approach described in the materials and methods. The expression of TM was scored using a semi-quantitative method, reflecting both the intensity of staining and the proportion of skin vessels that were affected, similar to methods used for biomarkers of cancer [37]. Scores were relative to normal TM staining in healthy skin from an unaffected individual, where TM is found abundantly in both keratinocytes of the epidermis and the endothelium of subcutaneous tissue ([38], see Fig 4A). This pattern and intensity of staining gained a maximum score of 9.

Using the strategy described, we investigated TM expression in lesions of 40 untreated, laboratory confirmed, BU patients (31 ulcers and nine plaque lesions; Table 1). Twenty five of the patients had Category 2 ulcers, however the cohort included also patients with Category 1 (8 patients) and Category 3 (6 patients). None of the patients had oedematous disease. In order to minimise topological variability between patients the location of punch sampling was standardised as far as possible (see Materials and Methods), and for ulcers was performed 1cm inside the outer margin of the induration surrounding the ulcer. In 32/40 lesions, histopathology revealed the presence of necrotic tissue, consistent with coagulative necrosis, within the biopsy. For SMA (Table 2), we found that it usually stained vessels both in the dermis and the subcutis (Fig 4B6 and 4B7). However, in four patients, SMA staining could not be detected in the subcutis, and these patients were therefore not considered in the TM analysis (Table 2). In comparison, CD31/PECAM-1 staining was fainter although it was still able to mark capillaries and small vessels in the skin (Fig 4B4 and 4B5).

**Table 1:** Clinical features of the BU patients from whom punch biopsy samples were analysed in this work.

		*M*. *ulcerans* confirmed (n = 40)
**Sex**	Male	24 (60%)
	Female	16 (40%)
**Age (years)**		11.5 (3–70)
**Type of lesion**	ulcerative	31 (77.5%)
	plaque	9 (22.5%)
**WHO category**	1	8 (20%)
	2	25 (62.5%)
	3	7 (17.5%)
**Location**	Upper extremities	22 (55%)
	Lower extremities	18 (45%)

The WHO category for each lesion was as follows: Category 1; a single small lesion or ulcer <5 cm in diameter, category 2; a single lesion 5–15 cm in diameter, category 3 a single lesion > 15 cm, multiple small lesions or lesions on the face. Data are n (%) or median (IQR).

**Table 2:** Histopathological features and biomarker expression in the skin of Buruli ulcer patients.

	Necrosis	Epidermis	Dermis					Subcutis				
	Appearance	TM	SMA	TM^1^			Fibrin	SMA	TM^1^			Fibrin
	n = 40	n = 40	n = 40	n = 40			n = 40	n = 40	n = 36			n = 40
	SCORE/3	SCORE/3	SCORE/3	Coverage/3	Intensity/3	SCORE/9	SCORE/3	SCORE/3	Coverage/3	Intensity/3	SCORE/9	SCORE/3
Expected value	0	3	1.5	3	3	9	0	1	3	3	9	0
Mean	1.73	1.6	1.838	2.04	1.35	3.47	1.13	1.5	1.36	0.94	2.15	2.19
SEM	0.19	0.16	0.11	0.15	0.16	0.54	0.14	0.2	0.18	0.17	0.52	0.14
Gaussian distribution?	N	N	N	Y	N	N	Y	N	N	N	N	N
Median	2	2	2	2	1	2	1	1.25	1	0.5	0.75	2.25
Range	0–3	0–3	1–3	0–3	0–3	0–9	0–3	0–3	0–3	0–3	0–9	0.5–3
25–75% Percentile	1–3	1–2.38	1–2.5	1–3	0.5–2	0.66–6	0.25–2	0.63–2	0.5–2	0.5–1.375	0.25–2	1–3
Major effect^2^	15 (37%)	8 (20%)	0 (0%)			26 (65%)^5^	3 (7%)	10 (25%)^6^			28 (78%)^7^	20 (50%)
Moderate effect^3^	17 (43%)	24 (60%)	35 (88%)			6 (15%)	27 (68%)	23 (58%)			3 (8%)	20 (50%)
No effect^4^	8 (20%)	8 (20%)	5 (12%)			8 (20%)	10 (25%)	7 (17%)			5 (14%)	0 (0%)

The distribution of the data were assessed using the D'Agostino & Pearson normality test with a cut-off value of P<0.05. Semi-quantitative scoring was carried out as described in Materials and Methods. TM is normally expressed in the keratinocytes of the epidermis and the endothelial cells of the dermis and subcutis. Necrosis was scored according to the appearance of the tissue in H&E staining.

^1^ TM staining was only scored in areas of lesions that had stained positively for SMA (endothelial cell marker), and a combined score derived from both the coverage and intensity was used.

^2^A major effect was defined as a score <1 for SMA and keratinocyte TM, <4 for endothelial TM combined score and >2 for fibrin staining and appearance of necrosis.

^3^A moderate effect was defined as a score of 1–2.5 inclusive for SMA and keratinocyte TM, combined endothelial TM score of 4–8 inclusive and 0.5–2 inclusive for fibrin staining and appearance of necrosis.

^4^No effect was defined as patients with maximum (SMA and TM) or zero (fibrin staining and appearance of necrosis) scores.

^5^In 11 (25%) of patients, TM was considered to be absent (combined TM score <1).

^6^Four of these patients did not show evidence of SMA staining in the subcutis and so TM was not quantified in these biopsies due to lack of intact endothelium.

^7^In 18 (50%) of patients, TM was considered to be absent (combined TM score <1)

Our analysis of TM expression revealed that, for most (but not all) BU lesions, TM abundance was reduced within the biopsy (Fig 4B and 4D). A major effect on the abundance of TM (defined as a combined score of less than 4 out of maximum of 9) was found in 78% of patient’s subcutis and 63% of patient’s dermis (Table 2, Fig 4B2 and 4B3 and 4D). Of these, TM was considered absent (combined score of <1) in either the subcutis or dermis of 21 lesions (53%). A further 15 and 22% showed a moderate reduction, respectively (Table 2). Therefore, the abundance of TM appeared to be more greatly affected in the subcutis than the dermis. The range in scores observed for ulcer and plaque category lesions were similar, suggesting that reduction of TM is a common feature of BU disease (Fig 4D).

In addition to changes to endothelial TM, we also observed changes to keratinocyte TM staining in the epidermis in some BU patients although this was more variable. In 8/40 (20%) samples TM could no longer be detected, whereas 24/40 (60%) showing moderate and 8/40 (20%) samples showing strong expression of TM in the hyperplastic regions (Fig 4C). As in endothelium, a similar range in scores was observed for both ulcer and plaque category lesions (Fig 4E), suggesting that changes to keratinocyte TM is also a common feature of BU disease. It should be noted that the wider variation in keratinocyte TM could reflect the location at which the punch biopsy was taken within the lesion. Indeed, there is a strong positive correlation between the score for dermal TM and epidermis TM (r = 0.693, p<0.0001) in these patients, suggesting that the two are linked.

### Abundant fibrin deposition in the infected skin of patients with Buruli ulcer

**Fig 5:**
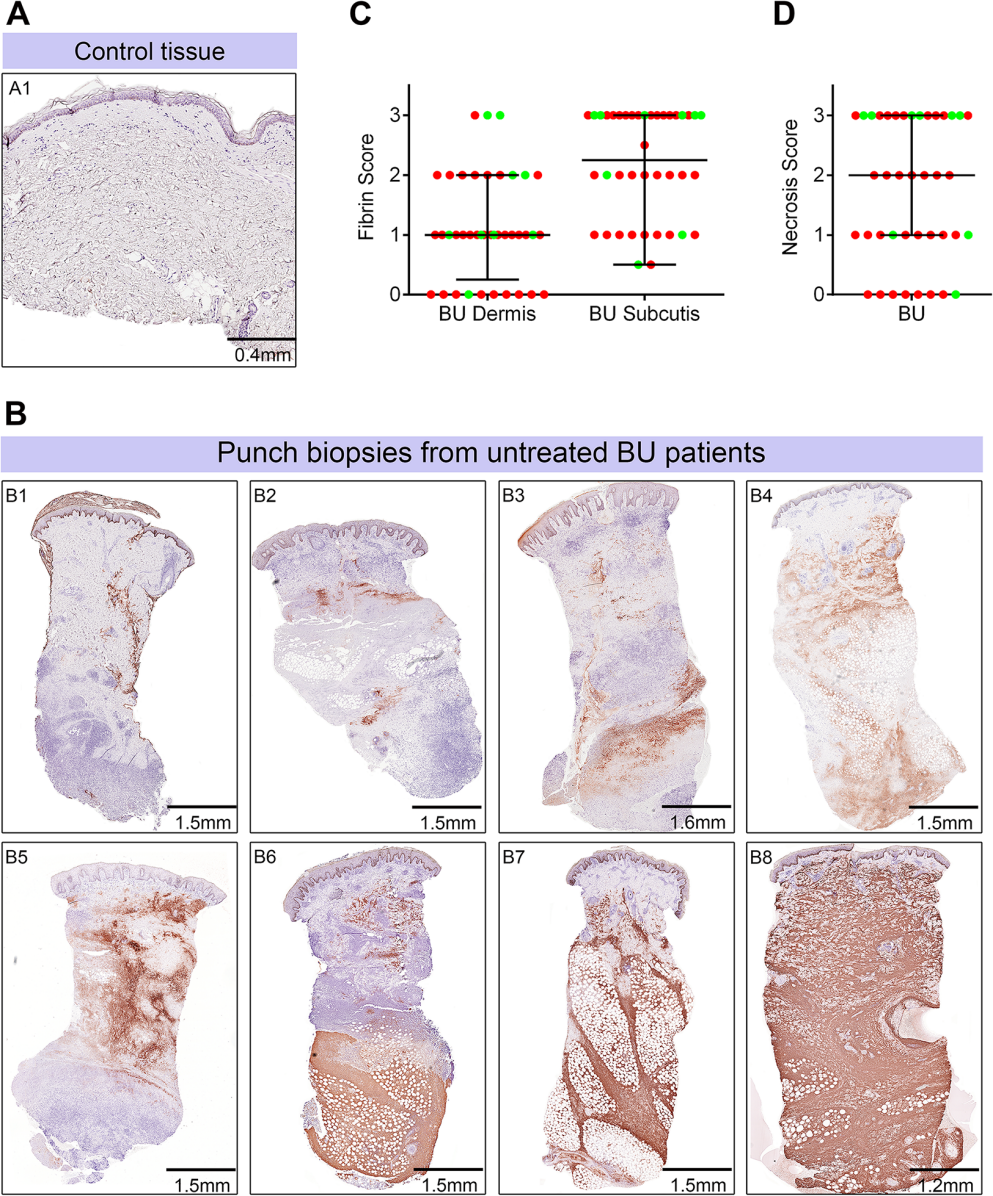
Abundant fibrin deposition in the skin of patients with Buruli ulcer. A and B. Histological sections were stained with an α-fibrin antibody and counterstained with Haematoxylin. Slides were analyzed with a DM2500 Microscope (Leica). Pictures were taken with an Aperio scanner. Comparative staining of a healthy skin sample from an unaffected donor (A) or 4mm punch biopsies from 8 different laboratory confirmed BU patients (B) showing the variability in fibrin staining observed ranging from small isolated fibrin depositions (B1-B2), to large deposition seen only in the dermis or subcutis (B3-B6) and finally to extensive depositions covering the whole tissue sample (B7-B8). C and D. Scoring was carried out as described in Materials and Methods and are relative to a maximum of 3. The score for each individual biopsy analysed is shown, consisting of 31 patients with ulcers (red circles) and 9 patients with plaque lesions (green circles). The expected score for healthy tissue was 0 for both fibrin and necrosis (see A). In all cases, error bars show the median and 25–75% percentile scores. C. Fibrin staining in the dermis and subcutis. D. The degree of necrosis in each biopsy was scored according to appearance of the entire biopsy.

It is well established that loss of TM in the presence of otherwise normal coagulation factors can cause fibrin deposition. While a single report from 1966 exists in the literature of fibrin deposition in BU lesions using phosphotungstic acid-haematoxylin stain (PTAH) [24], it is not known how widely occurring this phenomenon is. Immunohistochemistry analyses for fibrin deposition were therefore performed using a well-established monoclonal antibody that binds specifically to fibrin and not fibrinogen [39] (Fig 5 and Table 2). Semi-quantitative scoring was performed as for TM.

As expected, no fibrin deposition was observed in skin from a healthy individual, (Fig 5A, scored 0 out of 3). In contrast, lesions of all 40 BU patients that have been tested had fibrin deposited in the subcutis of the skin (Fig 5B), with 50% of them showing substantial deposition (scoring 2.5–3 out of 3). Furthermore, 75% of patients also had staining in the dermis although the effect was more moderate (Table 2 and Fig 5C). We found a clear positive correlation between the presence and degree of tissue necrosis (Fig 5D) with the amount of fibrin staining. This was in both the dermis (p = 0.0002) and subcutis (p = 0.0000002) (Table 3). Notably, subcutaneous tissue, where more fibrin is deposited, is the first skin tissue area to become necrotic in BU patients [40].

**Table 3:** Spearman’s correlation of histopathology/immunohistochemistry scoring.

				TM						
			Necrosis	coverage	intensity	SCORE	Fibrin		*Subcutis*	
	**Necrosis**	**r**	*	-0.187	-0.3070	-0.2304	0.7204	**r**	**Necrosis**	
		***P***	*	*0*.*275*	*0*.*0685*	*0*.*1765*	*<0*.*0001*	***P***		
	**coverage**	**r**	-0.100	*	0.8823	0.9690	-0.2560	**r**	**coverage**	
		***P***	*0*.*538*	*	*<0*.*0001*	*<0*.*0001*	*0*.*1319*	***P***		
**TM**	**intensity**	**r**	0.023	0.811	*	0.9581	-0.3821	**r**	**intensity**	**TM**
		***P***	*0*.*886*	*<0*.*0001*	*	*<0*.*0001*	*0*.*0214*	***P***		
	**SCORE**	**r**	-0.016	0.924	0.9648	*	-0.3111	**r**	**SCORE**	
		***P***	*0*.*920*	*<0*.*0001*	*<0*.*0001*	*	*0*.*0647*	***P***		
	**Fibrin**	**r**	0.557	-0.345	-0.1522	-0.2344	*	**r**	**Fibrin**	
		***P***	*<0*.*0001*	*0*.*029*	*0*.*3483*	*0*.*1454*	*	***P***		
	***Dermis***									

Spearman’s correlations and associated *P* value for dermis (bottom left of the table, below the cells marked ‘*‘) and subcutis (top right of the table, above the cells marked ‘*‘)

### Mycolactone’s effects on cellular thrombomodulin are not due to cell death

**Fig 6:**
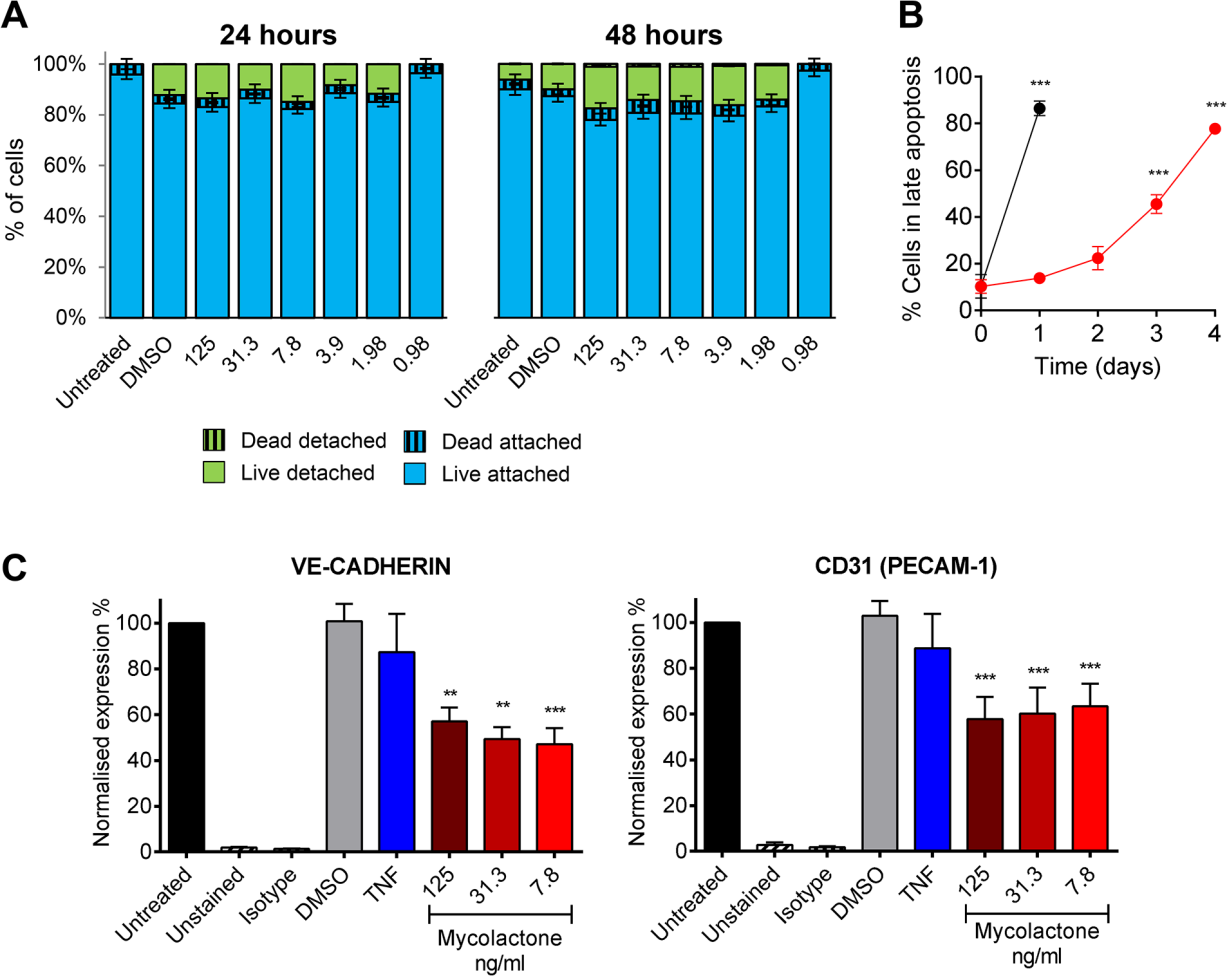
Mycolactone causes detachment of endothelial cells, apoptosis in 3–4 days, and depletes CD31 (PECAM-1) and VE-cadherin the surface. Human dermal microvascular endothelial cells were exposed to various concentrations of mycolactone (MYC), 10ng/ml TNF, 1μM staurosporin or 0.025% DMSO (solvent control) as appropriate. A. Both attached and detached cells were subjected to Calcein/EtBr staining for live/dead cells. The proportion of cells that were either attached or detached and alive (Calcein +/EtBr-), and attached or detached and dead (Calcein-/EtBr +) are expressed as a % of the total population of cells. mean±SEM, n = 3. B. HDMVECs were exposed to 7.8ng/ml mycolactone over a timecourse. Cells were then analysed by confocal microscopy following staining of cells with no-wash reagents. CellEvent caspase-3/7 green detection reagent identified cells undergoing apoptosis, alongside PI and DRAQ5. The number of cells in late apoptosis (positive for both active caspase 3/7 and PI) were counted in 3 fields and expressed as a proportion of total cells (DRAQ5 stained) Mean±SEM (n = 3). C. Cells were harvested and subjected to flow cytometry for VE-Cadherin and CD31 (PECAM-1). MFIs are expressed as % of untreated cells (mean±SEM, n = 4). Unstained and isotype bars are for untreated cells. **, P<0.01; ***, P<0.001.

Although reductions in TM abundance were found in live cells in patients (Fig 4) (as evidenced by their expression of SMA and CD31/PECAM-1 in nucleated cells), the link between fibrin staining and necrosis observed in the patients led us to investigate whether loss of TM is synchronised with cell death. Importantly, after 24 and even 48hrs of exposure to 7.8ng/ml mycolactone did not directly cause any significant loss of viability of the endothelial cells using a Live/Dead (calcein/Ethidium Bromide [EtBr] homodimer) assay (Fig 6A). After 24hrs ~20% of cells were detached from the plate, but were still alive when retrieved from the supernatant (Fig 6A). Therefore, the loss of TM from these cells appears to precede (or coincide with) changes in morphology, detachment or death. Indeed, when time to cell death was assessed using the CellEvent system (which does not require cells to be washed or manipulated in any way prior to analysis, therefore capturing attached, loosely attached and detached cells), significant numbers of HDMVEC did not enter late apoptosis until day 3, and the largest increase was observed between days 3 and 4, after which ~80% of the cells had died (Fig 6B).

Furthermore, since endothelial PECAM-1/CD31 staining was reduced in some BU lesions we investigated whether loss of adhesion molecules occurred *in vitro*, and might be linked to cell death. The impact of mycolactone on the expression of PECAM-1/CD31 and VE-cadherin was more variable, probably due to changes in turnover rates as the cells become confluent [41]. However, we found that both VE-cadherin and PECAM-1/CD31 were ~50% reduced by mycolactone exposure at 24hrs (Fig 6C).

## Discussion

The hallmark of BU disease is the presence of extensive subcutaneous necrosis, which is due to the cytotoxic activity of mycolactone [42]. However what is less clear is the earliest molecular event that triggers cellular death in *M*. *ulcerans* infected skin tissue. In the scientific literature to date there are many reports that have examined the cytopathic and cytotoxic effects of mycolactone *in vivo* and *in vitro*. For instance, TUNEL staining has been reported 2 days following injection of purified mycolactone in experimental animals as well as in BU patients [17,25]. More recently, conflicting data has been presented with more specific assays. While Bax and total caspase 3 and 9 have been detected in BU lesion [25], cleaved (i.e. activated, but possibly also unstable) caspase 3 was rarely detected in necrotic areas [43]. In this manuscript we provide compelling evidence that mycolactone-dependent depletion of TM occurs on HMDVEC and in diseased microvasculature, and that fibrin deposition is a common feature of BU lesions. Our findings suggest that the effect of mycolactone on tissue is probably both direct (due to cytotoxic/cytopathic effects on cells) and indirect, due to mycolactone-dependent loss of coagulation control and resultant tissue ischemia.

There are a number of potential mechanisms by which mycolactone might cause such a loss of TM. In the presence of thrombin, the thrombin-TM complex is internalised by endocytosis and unbound TM is returned to the cell surface [44]. Mycolactone is known to cause inappropriate actin polymerisation [32] that might credibly inhibit this process. However, whole cell lysate blots showed that TM did not accumulate in intracellular compartments, and depletion of TM was not restored by inhibition of WASP activity [32]. Instead, TM was found to deplete steadily from the cell surface due to an inability to replace TM during the normal protein turnover process, since newly synthesised, misolocalised, TM is degraded by the proteasome [18]. The rate of loss of TM was determined by flow cytometry (T_1/2_ 11.7±1.4hrs) and confocal microscopy of a recombinant GFP-tagged protein (T_1/2_ 8.5±0.6hrs). Both of these correspond well with the literature where human TM was shown to turn over at a rate of 5–10%/hour [45] (equating to a T_1/2_ of 5–10hrs). Notably other membrane proteins were less sensitive to mycolactone, presumably due to a lower turnover rate. For instance both the EPCR and PECAM-1 (CD-31) were only reduced by about 40–50% in 24hrs. Therefore TM's relatively higher turnover rate would appear to have rendered it more sensitive to mycolactone than other proteins.

Total deletion of TM is incompatible with life [46], whereas endothelial cell-specific deletion causes spontaneous thrombosis and a life-threatening coagulopathy including necrosis of the skin [47]. Conversely, a polymorphism in TM (THBD c.1418C>T) that increases surface TM due to decreased shedding, reduces the risk of deep vein thrombosis in carriers [48]. Furthermore, homozygous congenital deficiency of either protein S or protein C causes neonatal *purpura fulminans*; a combined disseminated intravascular coagulation and haemorrhagic disease that often presents with skin necrosis. Therefore, loss of the protein C anticoagulant pathway alone is sufficient to drive fibrin deposition and thrombosis. Indeed these pathways have been mechanistically linked in cerebral malaria [49] and sepsis [50]. There was not a complete correlation between fibrin deposition and TM abundance in the patients we examined in this study. Furthermore, in most patients, fibrin staining was observed predominantly in the tissue rather than the vessels, suggesting that other mechanisms also contribute to fibrin deposition such as reduced endothelial cell barrier function due to APC’s known cytoprotective effects [51,52]. However, it should be noted that fibrin deposited in vessels can be effectively cleared by the fibrinolytic system after a clotting event has taken place. A certain degree of caution is necessary due to the subjective nature of the immunohistochemistry scoring and the heterogeneity of BU skin lesions that presents a substantial challenge in quantification. However, qualitatively, strong fibrin and low thrombomodulin staining were frequently observed in BU lesions.

In this manuscript we have provided evidence that disruption of the protein C anticoagulant pathway due to mycolactone-dependent depletion of TM from endothelial cells is strongly associated with the fibrin deposition we observe in BU skin lesions. However, whether this is the direct cause of fibrin deposition is not easily answered in clinical samples from BU patients. This is because, at all clinical stages of disease, tissue necrosis is the defining feature. Indeed, there may be even vessel to vessel differences due to such factors as local mycolactone concentration gradients and local extent of necrosis. Thus it is not possible to determine the order in which TM depletion, fibrin deposition and death of skin tissue occurred in such samples.

There are alternative explanations for the fibrin deposition observed. Mycolactone reduced the expression of PECAM-1/CD31 and VE-Cadherin on the surface of endothelial cells. Loss of such adhesion molecules (including TM, itself a Ca^2+^-dependent adhesion molecule [53]) would increase the permeability of vessels. Indeed, endothelial cells are known to become ‘leaky’ in this way in many pathological states [54]. This might explain why fibrin deposition is frequently observed in BU lesions, since it would allow soluble proteins in the plasma to access this compartment even if erythrocytes or platelets cannot. Alternatively, the generation of high levels of Tissue Factor by dying cells or the exposure of collagen, von Willebrand factor (vWF) and other pro-coagulant surfaces should vessels be denuded would also promote fibrin deposition irrespective of TM. Depending on the concentration of mycolactone, and the duration of exposure (which will also vary throughout the evolution of the disease), different mechanisms may predominate. Since loss of TM precedes cell death by 2–3 days (at least *in vitro*), loss of coagulation control and resultant fibrin deposition/ischemia would be expected to precede cellular apoptosis caused directly by mycolactone. Therefore the promotion of a “procoagulant endothelial cell” is likely to be the one of the first triggers for disease at early (preclinical) stages of infection, as well as at the edges of the growing ulcer and encroaching mycolactone.

The expression of other ER-transiting components of the coagulation and anticoagulation systems may also be directly affected by mycolactone, and these are currently the subject of further ongoing investigations. The finding of fibrin deposition however, strongly suggests that the combined result of such effects is excessive thrombin generation. However, platelets do not seem to play a major role, since we found that mycolactone does not affect directly *in vitro* platelet function.

In addition to endothelial TM, keratinocyte TM [55] expression in the epidermis was also altered in punch biopsies from BU patients. Changes were variable and not as severe as in vessels. However loss or reduction of keratinocyte TM does appear to be a common feature of BU lesions and correlated with abundance of TM in the dermis. Heterogeneity may be related both to differences in the location from where tissue biopsies were taken and disease activity. Notably a keratinocyte-specific knockout of TM causes a substantial reduction in the ability to heal wounds [56] and is itself connected to the formation of cell-cell adhesion dependent on E-cadherin [57]. Any role for TM in wound healing would add to previously described mechanisms involving changes to actin dynamics, where mycolactone was shown to decrease migration of transformed epithelial cells (HeLa) in to a scratch wound *in vitro* [32].

In the literature there is a single anecdotal report of a patient with BU who was successfully treated by anticoagulant therapy (300mg heparin per day) alongside with rifampicin [58]. At the time, there was no supporting information to explain the success of this treatment and to date it has not lead to a change in treatment regimens, which depend primarily on rifampicin and streptomycin or clarithromycin [59]. However, our findings suggest that the use of anticoagulant support to go alongside antibiotic therapy might increase perfusion of the skin by decreasing fibrin deposition. This would improve the efficacy of antibiotic therapy, especially since mycolactone can persist in the skin (albeit at reduced levels) after sterilisation has been achieved [15].

In summary, in this manuscript we have shown that TM expression is highly sensitive to mycolactone exposure both *in vitro* and *in vivo*. We also demonstrated that fibrin deposition is a common feature of untreated BU lesions. The coagulation cascade is in a delicate balance, poised to prevent excessive blood loss due to injury whilst maintaining intravascular fluidity [50]. We therefore conclude that loss of coagulation control, driven in part by loss of TM, is strongly associated with the coagulative necrosis seen in BU.

## Materials and Methods

### Reagents

We used synthetic mycolactone A/B (kind gift of Prof. Yoshito Kishi, Harvard University) throughout these investigations [60]. Unless specified all other reagents are from Sigma-Aldrich.

### Cell culture and treatment

Primary human dermal microvascular endothelial cells (HDMVEC; LONZA) from two donors were used and cultured according to manufacturer’s recommendation in EGM-2MV (LONZA). Reagents for controls included DMSO (equivalent to top mycolactone dose; solvent control), 5μM protease inhibitor I (PSI), 10ng/ml TNF-α/IL-1β (Peprotech), 10μg/ml cycloheximide (CHX) and 5μg/ml human leukocyte elastase.

### Immunochemistry

Flow cytometry of live HDMVEC was carried out using standard techniques on a FACSCanto flow cytometer (BD Biosciences). Cells were detached with trypsin-EDTA (LONZA). Antibodies were from BD Pharmingen (EPCR; RCR-252, TM; 1A4, VE-Cadherin; 55-7H1, isotype controls) or Sigma (PECAM-1; WM-59). Western blot analysis was carried out using standard techniques, following lysis of cells with Gel sample buffer, separation by SDS-PAGE on 10% polyacrylamide gels and transfer to PVDF membranes. Antibodies were from Dako (TM; 1009) or Ambion (GAPDH). Soluble TM (sTM) was quantified in cell supernatants by ELISA (Stratech) according to the manufacturer’s protocol.

### Time-lapse confocal microscopy

Vector TMG was generated previously [53] and contains the full-length TM coding region cDNA upstream of eGFP. A control plasmid (expressing only eGFP) was prepared by removing the TM cDNA using BamHI and EcoRI restriction sites by standard cloning techniques. Both plasmids were stably transfected into HeLa cell lines by standard methodology and selection with G418. These cell lines are referred to as TM-GFP (transfected with vector TMG) and GFP (transfected with the GFP only plasmid). For confocal microscopy, cells were seeded into 6 well plates, treated as necessary and placed in a heated/humidified/5% CO_2_ chamber for 20hrs. Images were captured using in three different fields for each condition at 20min intervals using a Nikon A1 confocal laser scanning unit attached to an Eclipse Ti microscope. For quantitation, whole field fluorescence was calculated using NIS elements software (Nikon) with per-field background subtraction at each time-point and expressed as a percentage of starting fluorescence.

### Calibrated automated thrombography

The effect of mycolactone on thrombin generation was assessed by CAT according to a previously published method [61] using a Fluoroskan Ascent FL plate reader (Thermo Labsystem) in combination with Thrombinoscope software (Synapse BV). Thrombin generation was quantified in human pooled plasma containing various concentrations of mycolactone or 0.013% DMSO as a control. The reaction was initiated with 4pM tissue factor (Dade Innovin, Dade Behring), 4μM phospholipid vesicles (DOPS/DOPC/DOPE, 20:60:20) and 16.6mM CaCl_2_. Thrombin generation was monitored using 0.42mM of the fluorogenic substrate Z-GlyArg-AMC-HCl (Bachem).

### Platelet preparation, aggregation and activation assay

Human platelet preparation, aggregation and activation assays were performed according to well-established methods [62,63]. Briefly, blood was obtained from aspirin-free, healthy human volunteers with written informed consent as approved by the University of Reading Research Ethics Committee. Platelets were prepared and resuspended in modified Tyrodes-HEPES buffer (134mM NaCl, 2.9mM KCl, 0.34mM Na_2_HPO_4_, 12mM NaHCO_3_, 20mM HEPES and 1mM MgCl_2_, pH 7.3) and used at a final density of 2x10^8^cells/ml. Aggregation assays were performed as described, using collagen (Nycomed, Austria) in the presence or absence of either DMSO (final concentration 0.1%v/v) or mycolactone (at the indicated concentrations). Aggregation was quantified using an optical platelet aggregometer (Chrono-log, Havertown, PA) with continuous stirring (1200 rpm). Fibrinogen binding and P-selecting exposure were quantified by flow cytometry using FITC labelled rabbit anti-human fibrinogen antibody (Dako UK Ltd) and mouse anti-human CD62P antibody (BD Biosciences, UK), respectively. Washed platelets were incubated with mycolactone or DMSO as above before activation with CRP-XL (a selective agonist for platelet collagen receptor GPVI) at room temperature. The cells were fixed in 0.2% (v/v) formyl saline before analysis by flow cytometry. Data were analysed by calculating the median fluorescence intensity.

### Protein C activation assay

Protein C activation by cell surface-bound TM was quantified according to the method of Stearns-Kurosawa *et al* [36], with minor modifications. Purified human plasma proteins were obtained from Enzyme Research Laboratories. HDMVEC were washed three times with HBSS containing 3mM CaCl_2_, 0.6mM MgCl_2_ and 1% BSA (‘Activation Buffer’). Human protein C in Activation Buffer was added at the indicated concentration and protein C activation was initiated by the addition of 13.5nM α-thrombin. The cells were incubated for 30mins at 37°C at which point the reaction was stopped with 5μg antithrombin III and 3U heparin. In some cases, untreated cells were exposed to 50μg/ml of CTM1009, an anti-TM antibody known to inhibit protein C activation by preventing thrombin binding [36] for 30mins prior to the assay in HBSS containing 0.1% BSA and 0.02% sodium azide prior to washing as above. Activated protein C (APC) was quantified by assessing cleavage of the chromogenic substrate S-2366 (Chromogenix). Aliquots were transferred to 96-well microplates and S-2366 added at a final concentration of 200μM. The rate of substrate conversion was quantified at 405nm using a FLUOstar Omega plate reader (BMG LABTECH), and the concentration of APC interpolated from a standard curve of purified APC using Graphpad Prism v.6.

### Viability assays

For the viability assay using the LIVE/DEAD Viability/Cytotoxicity Kit (Molecular Probes Inc.), cells were washed twice with PBS incubated with 0.625μM CalceinAM and 10μM Ethidium homodimer-1 for 10min and then the plate was read on a FLUOstar Omega plate reader. During washing, the supernatants for retained and concentrated by gentle centrifugation in order to analyse the degree of cell detachment and the viability of those cells. Total live and dead cells were determined by the fluorescence of untreated or digitonin treated cells, respectively, considering both attached and detached portions. Live and dead cells were then expressed as a percentage of total cells.

In order to investigate apoptosis of cells, the CellEvent Caspase-3/7 green ready probes reagent was used. This fluorogenic indicator of activated caspase-3/7 can be used on live cells without washing, making it ideal for assessing mycolactone treated cells over longer time courses due to its cytopathic effects. After treatment, cells were stained with 0.3μg/ml PI (BD Biosciences, UK), 3μM DRAQ5 (Biostatus, UK) and 5μl CellEvent Caspase-3/7 green ready probes reagent (Invitrogen, Carlsbad, CA). Stained cells were observed by confocal microscopy using a Nikon A1 confocal laser scanning unit attached to an Eclipse Ti microscope. Active caspase-3/7 (green fluorescence), PI (red fluorescence) and nuclear (blue fluorescence) staining were analysed for 3 fields per treatment (minimum of 20 cells per field). Total cell numbers were determined by DRAQ5 staining, late apoptosis is expressed as a proportion of these cells which stained for both active caspase 3/7 and PI.

### Ethics statement

Ethical approval for analysing BU patient specimens was obtained from the National Ethics Committee of Cameroon, the Ethics Committee of the Heidelberg University Hospital, Germany, the Ethikkommission beider Basel, Basel, Switzerland and the provisional national ethical review board of the Ministry of Health Benin. Written informed consent was obtained from adult patients, or the guardians of patients who were children.

### Immunohistochemistry of punch biopsies

4mm BU punch biopsies were collected during other studies (ISRCTN72102977, IRB00006860 and EK.242/13) and were reanalysed for the current purpose. The clinical characteristics of the 40 untreated, laboratory reconfirmed, BU patients from whom biopsies were obtained are shown in (Table 1). In ulcerated BU lesions (31 patients) punch biopsies were taken 1cm inside the outer margin of the induration surrounding the ulcer. In contrast, from BU plaque lesions (9 patients) punch biopsies were collected from the non-ulcerated center of the lesion. After removal, tissue was fixed, transported, embedded into paraffin and cut into 5μm sections.

Immunohistochemistry was performed according to an established method [64]. Primary antibodies were from Dako (CD31; M0823, TM; M0617), Novocastra (SMA; NCL-SMA) Fibrin; 59D8 [39]). Biotin-conjugated secondary antibody was from Vectorlabs (BA2000). Staining was performed using the Vector ABC elite and the NovaRED system (Vectorlabs). Haematoxylin was used as a counter stain. Slides were analyzed with a DM2500 Microscope (Leica). Pictures were taken either with an Aperio scanner (Fig 4B1 and Fig 5) or with a Leica DFC 420 camera and the Leica application Suite V4 software. We have not been able to obtain 4mm punch biopsies from several healthy individuals, due to the invasive nature of this technique. Staining patterns and intensities for reference therefore included only one uninfected skin sample from a healthy individual. For further confirmation of TM and fibrin antibody staining performance in fixed tissues, we also examined additional samples from individuals with keloids and contact dermatitis.

Analysis of the immunohistochemistry results was not straightforward, due to the heterogeneity of skin and BU histopathology. This caused us to take a deliberately cautious approach to considering our findings. First, the integrity of vessels and endothelial cells within the biopsy tissue was assessed using the well-established markers smooth muscle alpha-actin (SMA) and CD31/PECAM-1, respectively. We scored each biomarker semi-quantitatively as follows; 0; no staining, 1; minimal staining, 2; medium staining and 3; strong staining. Scoring was performed separately for the epidermal, dermal and subcutaneous layer of the BU punch biopsies. Additionally, TM scoring was only carried out in regions of samples where vessels could be identified by SMA staining, in order to minimise the risk of scoring TM staining in vessels that had be destroyed by the infection. The semi-quantitative scoring for TM abundance incorporated both the intensity of staining and the extent of staining, according to the method of d’Adhemar [37], resulting in a maximum score of 9, which was equivalent to the staining observed in normal tissue. Fibrin staining and tissue necrosis had a maximum score of 3, but here normal tissue scored 0 for both biomarkers. Furthermore, in order to most accurately portray the trends and variations observed, the dermis and subcutis have been considered separately. We have not attempted statistical analysis of our results, due to the factors described above, but the scores are useful to indicate the range of findings we observed.

### Statistical analysis

Data were analysed using Graphpad Prism v.6 software. *In vitro* data was assessed using a one way ANOVA and Dunnett’s post-hoc test. Scoring data was assessed for a Gaussian distribution using D'Agostino & Pearson omnibus normality test. Since many data sets were not normally distributed, correlations used the method of Spearman.

### Accession numbers

β3 integrin: P05106, caspase 3: P42574, caspase 7: P55210, EPCR: Q9UNN8, fibrin (from fibrinogen α,β,γ): P02671, P02675, P02679, GAPDH: P16858, IL-1β: P01584, PECAM1/CD31: P16284, protein C: P04070, SMA: P62736, Thrombomodulin: P07204, TNF: P01375 ve-cadherin: P33151.

## Supporting Information

S1 Video The effect of DMSO on the expression of ER-transiting TM-GFP. HeLa cells were stably transfected with a plasmid encoding TM-GFP (a C-terminal fusion of human TM and GFP, therefore expressed on the membrane in an ER-dependent manner). Cells were exposed to 0.025% DMSO for 21hrs and fluorescence was captured by time-lapse microscopy at 20min intervals using a Nikon A1 confocal laser scanning unit attached to an Eclipse Ti microscope. DMSO was added to the wells before assembling the humidified chamber and setting up the experiment, therefore the first time point is approximately 1hr after reagent addition. One whole microscope field out of three per condition is shown. Videos were compressed in ImageJ and consist of 64 frames apiece and run at 6fps
(AVI)

S2 Video The effect of mycolactone on the expression of ER-transiting TM-GFP. HeLa cells were stably transfected with a plasmid encoding TM-GFP (a C-terminal fusion of human TM and GFP, therefore expressed on the membrane in an ER-dependent manner). Cells were exposed to 125ng/ml mycolactone for 21hrs and fluorescence was captured by time-lapse microscopy at 20min intervals using a Nikon A1 confocal laser scanning unit attached to an Eclipse Ti microscope. Mycolactone was added to the wells before assembling the humidified chamber and setting up the experiment, therefore the first time point is approximately 1hr after reagent addition. One whole microscope field out of three per condition is shown. Videos were compressed in ImageJ and consist of 64 frames apiece and run at 6fps
(AVI)

S3 Video The effect of DMSO on the expression of cytosolic GFP. HeLa cells were stably transfected with a plasmid encoding GFP alone (expressed in the cytosol). Cells were exposed to 0.025% DMSO for 21hrs and fluorescence was captured by time-lapse microscopy at 20min intervals using a Nikon A1 confocal laser scanning unit attached to an Eclipse Ti microscope. DMSO was added to the wells before assembling the humidified chamber and setting up the experiment, therefore the first time point is approximately 1hr after reagent addition. One whole microscope field out of three per condition is shown. Videos were compressed in ImageJ and consist of 64 frames apiece and run at 6fps
(AVI)

S4 Video The effect of mycolactone on the expression of cytosolic GFP. HeLa cells were stably transfected with a plasmid encoding GFP alone (expressed in the cytosol). Cells were exposed to 125ng/ml mycolactone for 21hrs and fluorescence was captured by time-lapse microscopy at 20min intervals using a Nikon A1 confocal laser scanning unit attached to an Eclipse Ti microscope. Mycolactone was added to the wells before assembling the humidified chamber and setting up the experiment, therefore the first time point is approximately 1hr after reagent addition. One whole microscope field out of three per condition is shown. Videos were compressed in ImageJ and consist of 64 frames apiece and run at 6fps
(AVI)

S1 Fig Mycolactone does not affect thrombin generation or platelet activation *per se*. A. Thrombin generation was measured by calibrated automated thrombography. Thrombin generation was quantified in human pooled plasma containing various concentrations of mycolactone or 0.13% DMSO as a control. The experiment was initiated with 4pM tissue factor, 4μM phospholipid vesicles, and 16.6mM CaCl_2_. Thrombin generation was monitored using 0.42mM of the fluorogenic substrate Z-GlyArg-AMC-HCl as described in the text. B. Human platelet activation was determined by quantifying fibrinogen binding and P-selectin exposure by flow cytometry and platelet aggregation. Washed human platelets were treated with various concentrations of mycolactone, 0.1% DMSO as a control or 0.5μg/ml CRP-XL (flow cytometry) or collagen (aggregation), and are expressed relative to CRP/collagen. Platelet aggregation was assessed using an optical platelet aggregometer. Mean±SEM n = 3.
(TIF)
