# Supplementary figures and images for: Mycolactone-Dependent Depletion of Endothelial Cell Thrombomodulin Is Strongly Associated with Fibrin Deposition in Buruli Ulcer Lesions
Source: PLoS Pathog. 2015 Jul 16;11(7):e1005011. doi: 10.1371/journal.ppat.1005011 (PMC4504485; doi:10.1371/journal.ppat.1005011)

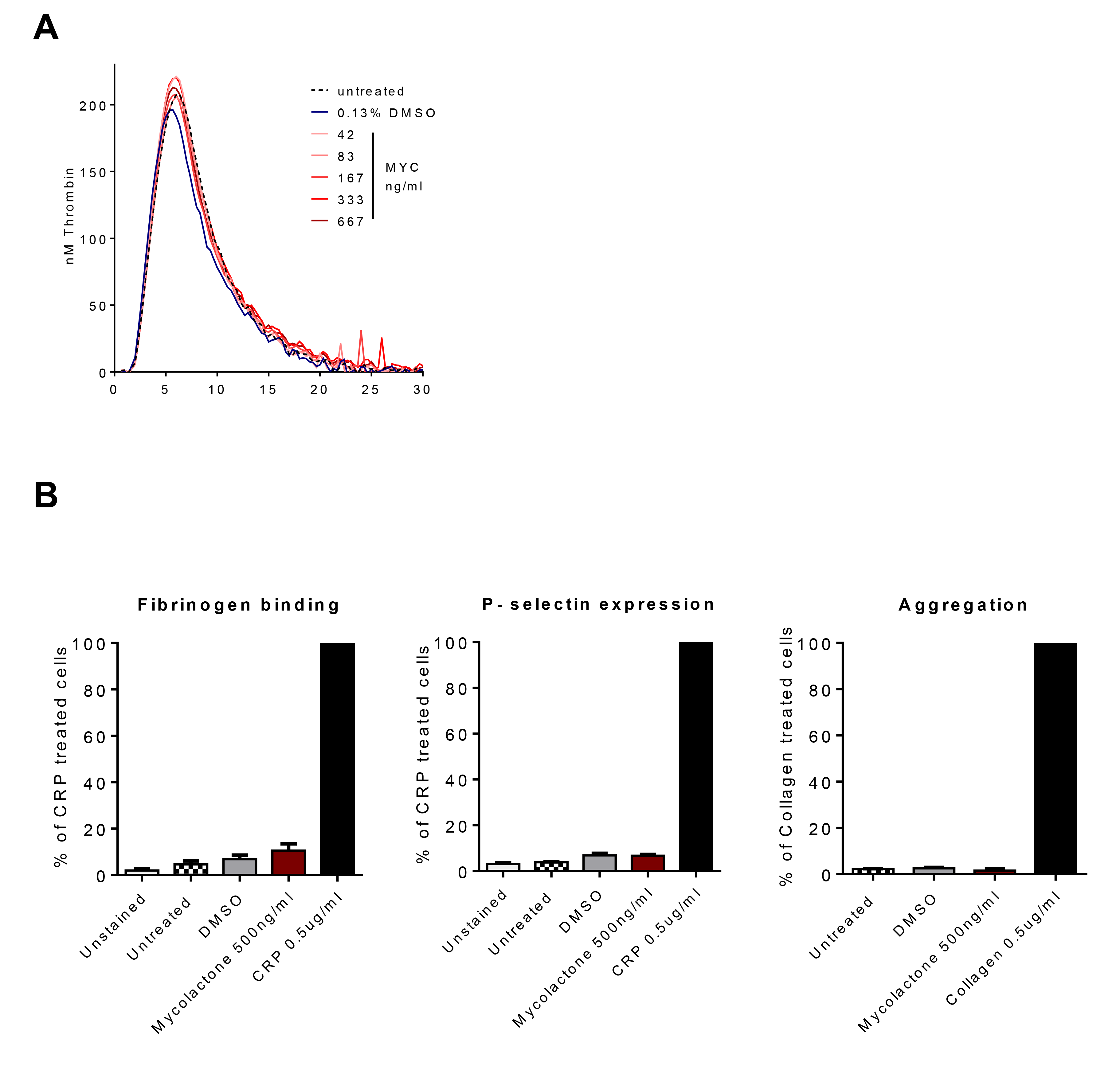

Supplement: S1 Fig — A. Thrombin generation was measured by calibrated automated thrombography. Thrombin generation was quantified in human pooled plasma containing various concentrations of mycolactone or 0.13% DMSO as a control. The experiment was initiated with 4pM tissue factor, 4μM phospholipid vesicles, and 16.6mM CaCl2. Thrombin generation was monitored using 0.42mM of the fluorogenic substrate Z-GlyArg-AMC-HCl as described in the text. B. Human platelet activation was determined by quantifying fibrinogen binding and P-selectin exposure by flow cytometry and platelet aggregation. Washed human platelets were treated with various concentrations of mycolactone, 0.1% DMSO as a control or 0.5μg/ml CRP-XL (flow cytometry) or collagen (aggregation), and are expressed relative to CRP/collagen. Platelet aggregation was assessed using an optical platelet aggregometer. Mean±SEM n = 3. (TIF) [file ppat.1005011.s005.tif]
